# Endomyocardial Fibrosis With Bilateral Ventricular Thrombus: A Case Report and Literature Review

**DOI:** 10.7759/cureus.45358

**Published:** 2023-09-16

**Authors:** Anas Mohamed, Abdelaziz Mohamed, Mustafa A. Al-Tikrity, Ahmed K Yasin, Nusiba Hafiz, Samah F Mohamed

**Affiliations:** 1 Internal Medicine, Hamad Medical Corporation, Doha, QAT; 2 Internal Medicine, Hamad General Hospital, Doha, QAT; 3 Pulmonary Medicine, Hamad Medical Corporation, Doha, QAT; 4 Radiodiagnosis, National Cancer Institute, Cairo University, Cairo, EGY; 5 Radiodiagnosis, Hamad General Hospital, Doha, QAT

**Keywords:** warfarin, septal hypertrophy, :heart failure, ventricular thrombus, endomyocardial fibrosis

## Abstract

Endomyocardial fibrosis (EMF) is a rare restrictive cardiomyopathy in non-tropical areas. It is seen in most of the patients living in or coming from tropical areas, and is rarely seen in patients who have never visited these areas. It is characterized by fibrotic thickening of the endocardium, predominantly affecting the ventricular apices and inflow tracts. Although thrombus formation is a known complication in various cardiac conditions such as atrial fibrillation, atrial flutter, ventricular heart disease, and patent foramen ovale, the occurrence of bilateral thrombus in EMF is exceptionally rare. We present a case report describing a unique finding of bilateral ventricular thrombus in a patient diagnosed with EMF, highlighting the clinical presentation, diagnostic approach, and management challenges associated with this rare phenomenon.

## Introduction

Endomyocardial fibrosis (EMF) is a cardiac condition characterized by subendocardial fibrosis, primarily affecting the apex and inflow tracts of one or both ventricles [1].

Although commonly observed in tropical areas, there have been sporadic cases reported in individuals with no prior exposure to these regions.

The clinical presentation of EMF is predominantly marked by left ventricular filling restriction and atrioventricular valve regurgitation, attributed to the involvement of papillary muscles and chordae tendinae. These pathological changes eventually lead to the development of heart failure [1,2].

The diagnosis of EMF relies on a combination of the patient's medical history, symptoms suggestive of heart failure, and echocardiographic findings indicative of restrictive ventricular filling and atrioventricular valve regurgitation. However, the exact etiology and pathogenesis of EMF remain largely unknown, although several potential triggers, such as malnutrition, toxins, infectious agents, allergens, and hyper-eosinophilia, have been proposed based on research findings [1,3].

In this case report, we present a unique instance of EMF, highlighting the diagnostic approach, clinical manifestations, and the challenge of complications along with management. 

## Case presentation

A 47-year-old Nigerian lady with a past medical history of dyslipidemia, managed with medications, presented with a complaint of intermittent chest discomfort and mild exertion during her daily activities over the past two years. She had previously undergone a CT cardiac coronary angiogram, which yielded normal results.

During her routine outpatient follow-up, echocardiography revealed dilation of the right ventricle (RV) associated with reduced ventricular compliance and a decrease in right ventricle function without evidence of pulmonary hypertension, left heart failure, or tricuspid valve disease, which are the most common causes of right ventricle dilation. Simultaneously, there appears to be a need for enhanced delineation of both the structural and functional aspects of the heart muscle, which cannot be discerned through echocardiography alone. Because the chest discomfort symptoms persist, it was decided to conduct a cardiac MRI for a more detailed evaluation of the right ventricle. This choice was made because of the accessibility of cardiac MRI in our hospital, which offers high-resolution imaging, improved tissue characterization, and a comprehensive assessment of both cardiac anatomy and function. This decision led to the determination to proceed with the cardiac MRI rather than contrast echocardiography. 

**Figure 1:**
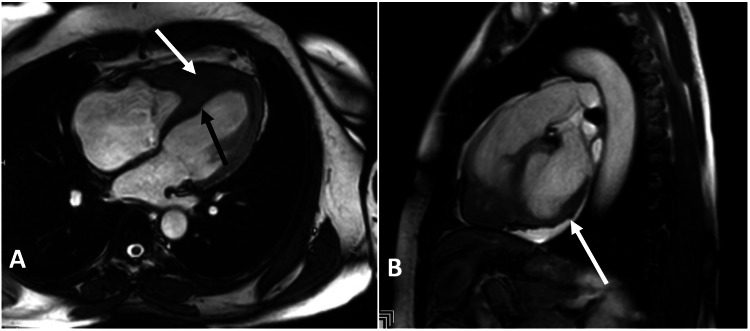
Cardiac MRI, CINE 4 chamber clip (A), and CINE RVOT clip (B) show mid-to-apical LV septal hypertrophy (black arrow) along with RV wall thickening (white arrows). LV: left ventricle, RV: right ventricle

**Figure 2:**
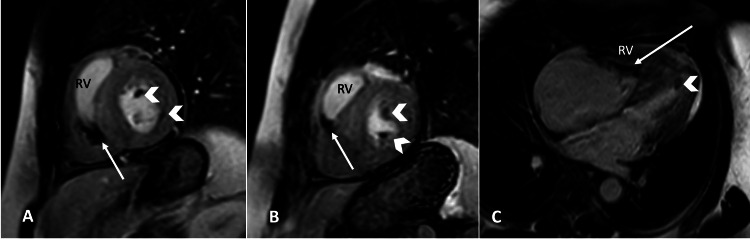
Early gadolinium-enhanced images in the short axis at the mid-cavity level (A and B) and four chamber views show signal-void non-enhancing thrombi in the right ventricle (RV [white arrows]) and left ventricle (white arrow heads).

The cardiac MRI revealed infiltrative heart disease primarily affecting the right ventricle, causing wall thickness and mid-to-apical LV septal hypertrophy (Figure 1), accompanied by the presence of bilateral small ventricular thrombi (Figure 2). Our initial differential diagnosis was amyloidosis, causing infiltration of the heart muscle, cardiac sarcoidosis, and endomyocardial fibrosis. 

Consequently, the patient was admitted as an inpatient to initiate anticoagulation therapy and conduct further investigations.

A transthyretin cardiac amyloidosis study was performed using an IV injection of 99 mTc-PYP, followed by static images and SPECT/CT to investigate the possibility of cardiac amyloidosis. The results indicated no evidence of amyloidosis. The case was re-discussed again with another radiologist, and the history and background of the patient, in addition to the MRI findings, did not go with cardiac sarcoidosis.

Due to a lack of sufficient data regarding the use of anticoagulation for this condition and considering the observed resistance to anticoagulation therapy according to expert advice, the patient was initiated on a treatment regimen involving warfarin and enoxaparin for bridging therapy. She was discharged from the hospital once the appropriate therapeutic range for warfarin was attained. The patient was advised to have follow-up appointments with the cardiology department as an outpatient after three months to check for INR level and a possible repeat of the cardiac MRI to assess for resolution of thrombi. Meanwhile, the patient is following routinely in the anticoagulation clinic for routine checking of INR level. 

## Discussion

Endomyocardial fibrosis (EMF) is a medical condition initially recognized in Uganda in 1947. It predominantly occurs in less economically developed areas, including regions in Africa, Asia, and South America, particularly in tropical and subtropical zones. This condition is seldom encountered in developed nations since the majority of affected individuals either reside in or originate from these geographical regions [2].

Typically, EMF is associated with parasitic infections and is a significant cause of heart failure in tropical and sub-tropical areas [3,1].

However, in our case, the patient had been living in a non-tropical area for over a decade with no risk factors for parasitic infections. Additionally, the patient did not exhibit eosinophilia or autoimmune abnormalities. While EMF cases are predominantly reported in tropical areas, there have been some cases reported in non-tropical regions. These cases have been linked to conditions such as vasculitis (particularly Churg-Strauss syndrome), malignancy, connective tissue disorders, or allergies [3,2].

However, our patient had no evidence of autoimmune diseases, allergies, or any significant medical history.

In contrast to many EMF cases that present with complications such as heart failure and arrhythmias, our patient's primary symptom was on-and-off chest discomfort associated with exertion over a period of two years, most likely due to reduced blood flow to the heart muscle, which was evident by the findings in the echocardiography and cardiac MRI, which showed clear restrictions in the heart muscle. At the same time, the patient underwent a CT coronary angiogram, which did not reveal any significant coronary artery disease.

Thrombosis is one of the common complications of EMF, and in our case, biventricular small thrombi were discovered during cardiac MRI evaluation. The right ventricle thrombi were adherent to the apical inferior wall, which is the largest, measuring about 2.5 cm; the left ventricle thrombi were adherent to the apical inferior segment and the epical cap, with the largest measuring approximately 1.3 cm. While eosinophilia and eosinophilic infiltrates have been associated with thrombotic events in some cases, our patient did not exhibit these features. There were also no other predisposing factors for thrombosis, such as recent surgery in the extremities, hormonal therapy medications, genetic hypercoagulabilities like prothrombin gene mutation, factor V leiden, antithrombin deficiency, protein C deficiency, and protein S deficiency, or any evidence of advanced atherosclerosis. The patient's only identified risk factor was dyslipidemia [4]. 

Management of EMF is challenging due to limited evidence-based guidelines. Standard medical therapy includes diuretics for decongestion, ACE inhibitors/ARBs, and beta-blockers for symptomatic relief. Anticoagulation is recommended for patients with a confirmed endocardial thrombus [5].

Surgical intervention, specifically endocardectomy combined with valvular replacement, is utilized in advanced cases. However, this procedure carries a high mortality rate, and recurrence after surgery has been reported.

In severe and treatment-resistant cases, heart transplantation may be the last resort [6].

## Conclusions

Bilateral thrombus formation in the context of endomyocardial fibrosis is an exceedingly rare occurrence. This case report highlights the need for heightened awareness and consideration of thrombotic complications in patients diagnosed with EMF. Early detection, accurate diagnosis, and appropriate management are essential to improving clinical outcomes and preventing potentially life-threatening events.

Further research and case reports are warranted to enhance our understanding of the pathogenesis, clinical implications, and optimal management strategies for this unusual finding in EMF.
